# Peripheral blood cells from children with RASopathies show enhanced spontaneous colonies growth *in vitro* and hyperactive RAS signaling

**DOI:** 10.1038/bcj.2015.52

**Published:** 2015-07-17

**Authors:** G Gaipa, C Bugarin, P Cianci, J Sarno, P Bonaccorso, A Biondi, A Selicorni

**Affiliations:** 1 M. Tettamanti Research Centre, Pediatric Clinic, University of Milano-Bicocca, Monza, Italy; 2 Pediatrics Clinical Genetics, MBBM—AO San Gerardo Foundation, University of Milano-Bicocca, Monza, Italy; 3 Center of Pediatric Hematology Oncology, Azienda Policlinico-OVE, University of Catania, Catania, Italy

## Abstract

Germline mutations in genes coding for molecules involved in the RAS/RAF/MEK/ERK pathway are the hallmarks of a newly classified family of autosomal dominant syndromes termed RASopathies. Myeloproliferative disorders (MPDs), in particular, juvenile myelomonocytic leukemia, can lead to potentially severe complications in children with Noonan syndrome (NS). We studied 27 children with NS or other RASopathies and 35 age-matched children as control subjects. Peripheral blood (PB) cells from these patients were studied for *in vitro* colony-forming units (CFUs) activity, as well as for intracellular phosphosignaling. Higher spontaneous growth of both burst-forming units-erythroid (BFU-E) and CFU-granulocyte/macrophage (CFU-GM) colonies from RAS-mutated patients were observed as compared with control subjects. We also observed a significantly higher amount of GM-colony-stimulating factor-induced p-ERK in children with RASopathies. Our findings demonstrate for the first time that PB cells isolated from children suffering from NS or other RASopathies without MPD display enhanced BFU-E and CFU-GM colony formation *in vitro*. The biological significance of these findings clearly awaits further studies. Collectively, our data provide a basis for further investigating of only partially characterized hematological alterations present in children suffering from RASopathies, and may provide new markers for progression toward malignant MPD in these patients.

## Introduction

The presence of germline mutations in genes coding for molecules involved in the RAS/RAF/MEK/ERK mitogen-activating protein kinase (MAPK) cascade is the molecular basis for a newly classified family of autosomal dominant syndromes termed ‘RASopathies'. These disorders that include Noonan syndrome (NS), LEOPARD syndrome, Costello syndrome, cardiofaciocutaneous syndrome, neurofibromatosis 1 and Legius syndrome are developmental syndromes affecting ~1 in 1000 live birth.^1, 2, 3^ Each of them exhibits distinctive phenotypic features, although there are numerous overlapping clinical manifestations including dysmorphic craniofacial features, congenital cardiac defects, skin, skeletal and ocular abnormalities, varying degrees of intellectual disability and increased cancer risk.^4, 5, 6^ NS, the most common among these disorders, is characterized by heterozygous germline mutation in the *PTPN11* gene in approximately half of the cases.^7^ The *PTPN11* proto-oncogene encodes Src-homology tyrosine phosphatase-2 (SHP-2), a protein tyrosine phosphatase with a role in signal transduction and hematopoiesis.^8^

Progression toward myeloproliferative and myelodysplastic disorders, which can cause severe medical problems, may occur in NS patients, although spontaneous remission has often been described.^9, 10, 11^ Juvenile myelomonocytic leukemia (JMML) and JMML-like myeloproliferative disorder may also occur in some NS patients with favorable outcome.^12^ JMML is an aggressive myeloproliferative neoplasm of childhood characterized by uncontrolled proliferation of monocytic and granulocytic cells. JMML-derived hematopoietic precursor cells often show hypersensitivity to granulocyte-macrophage colony-stimulating factor (GM-CSF) *in vitro*.^13, 14^ PTPN11 somatic missense mutations have been identified in approximately one-third of JMML cases. In this regard, analysis of the mutational spectra observed in NS vs JMML patients clearly indicates that germline PTPN11 NS-associated mutations have only a mild effect on development and hematopoiesis when compared with JMML-associated somatic PTPN11 lesions.^15, 16, 17, 18, 19^

Although the molecular mechanism underlying the progression of hematopoietic disorders observed in patients bearing PTPN11 mutations is not fully understood, several *in vitro* and *in vivo* studies indicate that hyperactivation of the tyrosine phosphatase SHP-2, encoded by *PTPN11*, may have a role in aberrant activation of both the JAK/STAT and RAS/MAPK/ERK signaling pathways favoring the pathogenesis of NS and JMML.^20, 21, 22^

On the basis of these findings, here we have systematically evaluated the extent of RAS/MAPK signaling activation in a cohort of RAS-mutated patients in order to explore the effect of aberrant RAS activation in circulating progenitors and mature monocytes, and possibly to determine genotype–phenotype correlations.

## Materials and Methods

### Patients and samples

In this study, a total of 27 patients, admitted to Pediatrics Clinical Genetics of San Gerardo Hospital, were enrolled with a clinical diagnosis of NS or related disorder (hereinafter referred to as RAS-mutated patients). As control cohort, we examined 35 age-matched children, which included both patients without onco-hematological diseases and patients with others genetic defects that could not affect the JAK/STAT and RAS/RAF/ERK pathways. Peripheral blood (PB) cells (*n*=22) were cultured *in vitro* in order to evaluate both spontaneous and growth factor-stimulated colony-forming unit (CFU) activity. In addition, PB monocytes were assessed for IL (interleukin)-6 (50 ng/ml)-induced response of p-STAT3 (*n*=15), GM-CSF (0.1 and 10 ng/ml)-induced response of p-STAT5 (*n*=15) and p-ERK (*n*=16) by phosphoflow cytometry, as previously described.^23^ Clinical and biological characteristics of the patients enrolled in this study are shown in Table 1. This study was approved by the local institutional ethics committees and carried out with the informed consent of the patients' guardians.

### Cytokine stimulation and phospho-specific flow cytometry

Freshly isolated PB mononuclear cells were suspended in serum-free media *ex vivo* at a concentration of 1–2 × 10^6^ cells per ml, rested for 1 h at 37 °C, and assessed for total cell count and viability by trypan blue dye exclusion before being subject to phosphoflow assay. Samples were then stimulated with 0.1 and 10 ng/ml of GM-CSF or 50 ng/ml of IL-6 for 10 and 15 min, respectively, to allow signal transduction. Cells were then fixed with paraformaldehyde and permeabilized with ice-cold methanol according to published methods^23^ and then incubated with anti-phosphoprotein-directed monoclonal antibodies, or isotype-matched IgG, and surface antigen-directed monoclonal antibodies, previously tested for their maintenance of expression after fixation and permeabilization.

**Figure 1:**
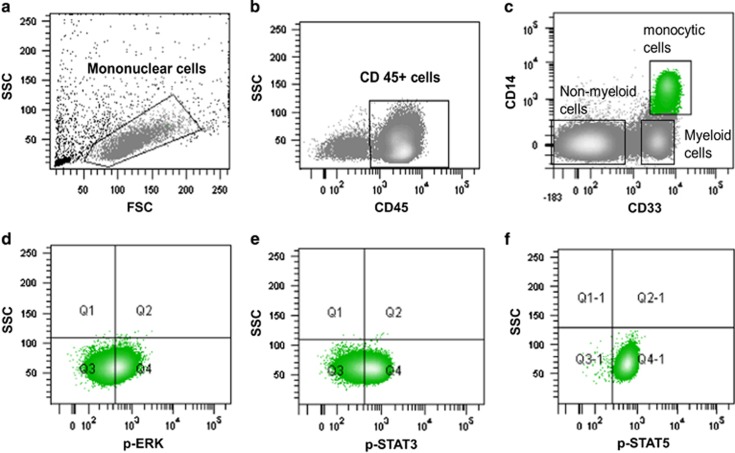
Mononuclear cells were identified on the basis of physical parameters (**a**) and then selected according to their CD45 reactivity (**b**). Within the CD45+ cells, monocytes (in green), non-myeloid and myeloid cells could be distinguished by means of their CD33 and CD14 reactivity (**c**). Phosphoprotein expression was then measured on monocytic gated cells (**d**–**f**).

Samples were stained with antibodies such as CD33 PE (clone P67.6), CD14 APC-H7 (clone MϕP9), CD45 PerCP (clone 2D1), p-STAT5 Alexa 647 (pY694), p-STAT3 Alexa 488 (pY705), p-ERK1/2 Alexa 488 (T202/Y204) or isotypes IgG1k Alexa 488 and Alexa 647 (clone MOPC-21) (BD Bioscience, San José, CA, USA), and data were acquired on a FACSaria flow cytometer (BD Bioscience). At least 100 000 events were collected and analyzed using DIVA software (San José, CA, USA). Basal levels of each phosphoprotein were calculated as percentage of phospho-positive cells in unstimulated conditions. Response to stimulation upon cytokine treatment (for example, recombinant human GM-CSF or IL-6) was calculated by subtracting the percentage of unstimulated phospho-positive cells under basal conditions. Each phosphoprotein reactivity was evaluated in CD14+/CD33+/CD45+ cells as assessed by the flow cytometric gating strategy described in Figure 1. GM-CSF-induced p-STAT5 signaling in monocytic cells contained was considered as an internal positive control of functional signaling.

### Spontaneous colony growth assay

Freshly isolated PB mononuclear cells were cultured at a density of 5 × 10^5^/ml in methylcellulose medium (Methocult H4230, Stem Cell, Vancouver, BC, Canada) in the presence or absence of 10% conditioned medium from the 5637 tumor cell line and 2 U/ml erythropoietin. Spontaneous and stimulated growth of CFU-GM and BFU-E was determined after 14–21 days of incubation as previously described.^24^

### Statistics

The unpaired *t*-test (*P*-value) was used to compare the values of both CFU-GM and BFU-E growth between RAS-mutated patients (*n* 27) and control subjects (*n* 35), as well as to compare p-STAT3 and p-ERK values between the same two groups. All statistical tests were performed at a significance level of *P*<0.05 (two-tailed).

## Results

### Clonogenic assay

**Figure 2:**
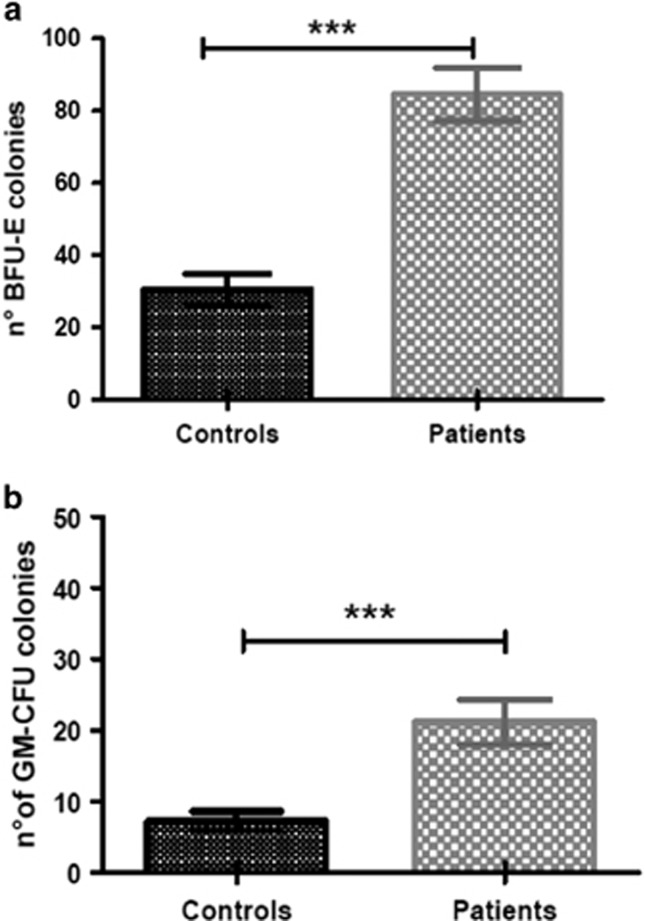
Representative quantitative BFU-E (**a**) and CFU-GM (**b**) colony numbers as assessed by clonogenic assays. *t* test, ***P<0.001.

Clonogenic assays from PB precursor cells showed a significantly higher spontaneous growth rate of both BFU-E and CFU-GM in RAS-mutated patients (*n*=22) than control subjects (*n*=22). We observed an increased number of BFU-E colonies (mean±s.e.m.): 84.45±7.37% vs 30.50±4.32%, *P*<0.0001, as well as enhanced growth of CFU-GM, (21.14±3.15% vs 7.23±1.38%, *P*=0.0002; Figures 2a and b, respectively). Augmented colony formation activity of RAS-mutated cells was also observed upon incubation in growth factor-supplemented medium: BFU-E (78.41±6.60% vs 31.14±4.80%, *P*<0.0001) and CFU-GM (21.59±2.93% vs 8.95±1.77%, *P*=0.0006).

### Phosphoflow assay

**Figure 3:**
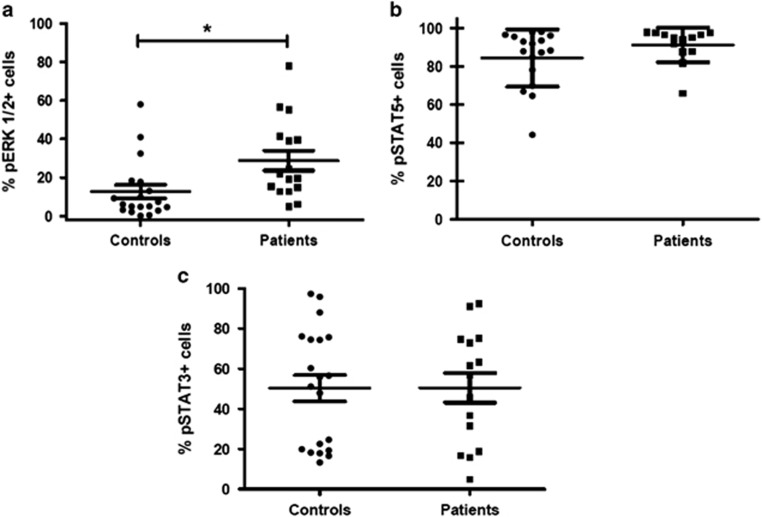
Scatter diagram of p-ERK+ cells treated with 10 ng/ml GM-CSF (**a**), p-STAT5+ cells treated with 0.1 ng/ml GM-CSF (**b**) and p-STAT3+ cells treated with 50 ng/ml IL-6 (**c**). Data are shown as percentages with mean (central bars) and ranges (whiskers). *t* test, *P<0.05.

A significantly enhanced amount of GM-CSF-stimulated p-ERK-positive cells was observed in monocytes isolated from RAS-mutated patients (*n*=16) as compared with control subjects (*n*=19): 28.84±5.192% vs 12.74±3.52% (mean±s.e.m.) *P*=0.0128, respectively (Figure 3a). This enhanced p-ERK response was also observed at lower dose of GM-CSF (0.1 ng/ml) (4.13±2.34% vs 0.28±0.15%) or under basal conditions (3.39±0.94% vs 1.42±0.54%) in RAS-mutated patients (*n*=9) as compared with control subject (*n*=12), although these differences were not statistically significant. When we tested the GM-CSF-induced p-STAT5 response in monocytic cells, we failed to find significant differences between patients (*n*=13) and control subjects (*n*=17): 91.15±2.5% vs 84.39±3.63% (mean±s.e.m.) at 0.1 ng/ml GM-CSF and 96.65±0.62% vs 96.91±0.49% at 10 ng/ml GM-CSF (Figure 3b). Finally, IL-6-stimulated p-STAT3 levels were also measured in monocytes from patients (*n*=15) and controls (*n*=20). Both subgroups responded to such stimuli in a similar fashion (mean±s.e.m. at 50 ng/ml IL-6: 50.49±7.38% vs 50.30±6.55%, *P*=NS; Figure 3c).

## Discussion

It has been shown that individuals with NS are at increased risk of developing a myeloproliferative disorder (NS/MPD) that may resolve spontaneously or progress to JMML.^25, 26, 27^ neuro cardio facial cutaneous syndromes, in particular NS, are related to JMML in terms of both germline and somatic mutations of genes involved in the RAS/MAPK signaling pathway.

Here we demonstrate for the first time that RAS-mutated non-JMML patients are characterized by a significantly higher spontaneous growth rate, *in vitro,* of both BFU-E- and CFU-GM-circulating progenitors than control subjects. This observation is in good agreement with previous studies using mice carrying germline mutations of PTPN11 (for example, D61Y, E76K and D61G), which showed an increased number of BFU-E and GM-CFU colonies with aberrant differentiation and MAPK-induced GM-CSF hyperactivation.^25, 26, 28^ Such enhanced growth of circulating hematopoietic progenitors, which we have consistently observed in our cohort of RAS-mutated patients, although not to the same extent of that measured in JMML patients, may reflect a constitutional predisposition to cell hyperproliferation of subjects, such as NS patients, who display a moderately activated RAS/MAPK signaling pathway.

Timeus *et al.*^29^ have recently reported a significant decrease in the apoptotic rate of circulating CD34+ hematopoietic progenitors in NS patients suggesting an increased CD34+ cell survival, despite the lack of evidence of an abnormal pattern in both CD34+ cells and CFU-GMs. Thus, this evidence supports a model whereby NS patients displaying aberrant behavior of hematopoietic progenitors are predisposed to developing NS/MPD or JMML.

Mutations of PTPN11 have been shown to promote RAS activation, which in turn induces activation of the Raf/MEK/MAPK cascade.^30, 31^ Here we demonstrate that such activation can be consistently evaluated in RAS-mutated patients by simply measuring p-ERK levels through accurate single-cell phosphoflow analysis. Thus, our findings seem to indicate that the extent of ERK phosphorylation in hematopoietic progenitors might be a potential new marker for diagnosis and monitoring of NS patients at risk of developing MPDs.

Some authors have hypothesized that Shp-2-mediated STAT3 hypophosphorylation might also have a role in the phenotypic abnormalities observed in NS and/or during the pathogenesis of JMML.^22^ Contrary to the results by Zhang *et al.*, we did not observed any altered pattern of STAT3 phosphorylation in our series of PB cells from RAS-mutated patients. However, we could not measure p-STAT3 on circulating CD34+/CD33+ myeloid progenitors due to the low number of such cells suitable for phosphoflow analysis. Thus, further studies are clearly needed to shed light on the role played by JAK/STAT signaling in NS/MPDs.

Finally, in good agreement with previous data in JMML patients,^23^ we could not detect any significant difference between the constitutive STAT5 phosphorylation in RAS-mutated patients vs control subjects.

These data together with the findings by Kotecha et *al.*,^32^ who failed to detect an increase in p-STAT5 in NS patients, strongly suggest that lack of p-STAT5 hyperactivation may be related to the hematological benign course of most syndromic cases with Ras mutations.

Recently, Strullu *et al.* have reported a large prospective cohort of NS patients where ~3% of them met the consensus diagnostic criteria for JMML, and nearly half of them showed severe neonatal manifestations and died within the first month of life.^33^ As the incidence of childhood RASopathies and MPDs may be underestimated due to such early lethality, the authors recommended that all cases of childhood NS should be systematically evaluated for clinical signs of MPD and basic hematological parameters during the first year of life.^33^ In this regard, our work provides new insights into the molecular characterization of children with NS that could potentially be exploited for more advanced screening and monitoring of children with RASopathies and MPDs.

Taken together, our results allow us to hypothesize the existence of a ‘hematological phenotype'—in non-JMML NS or RAS-mutated syndromic patients—that is secondary to germline RAS mutations and able to induce a mild activation of the RAS/MAPK cascade. Further studies are needed to fully understand the role of such aberrant patterns during the pathogenesis of hematological diseases in these patients.

## Figures and Tables

**Table 1:** Clinical and laboratory characteristics of 27 RAS-mutated patients enrolled from October 2011 to March 2014

*Characteristic*	*Number*	*%*	*Mean*	*Range*
Patients	27	100	—	—
				
*Gender*				
Male	18	66.7	—	—
Female	9	33.3		
Age (months)			110	12–264
				
*Phenotype*				
NS	22	81.5	—	—
LS	3	11.1		
CFC	2	7.4		
				
*Genotype*				
PTPN11	23	85.2	—	—
MEK	2	7.4		
BRAF	1	3.7		
SHOC2	1	3.7		
				
WBC (10^9^/l)	—	—	8.75	4.76–23.05
Monocytes (10^9^/l)	—	—	0.78	0.43–1.55

Abbreviations: CFC, cardiofaciocutaneous syndrome; NS, Noonan syndrome; LS, LEOPARD syndrome; WBC, white blood cell.
